# PKCα replaces AMPK to regulate mitophagy: Another PEDF role on ischaemic cardioprotection

**DOI:** 10.1111/jcmm.13849

**Published:** 2018-09-19

**Authors:** Haoran Miao, Fan Qiu, Bing Huang, Xiucheng Liu, Hao Zhang, Zhiwei Liu, Yanliang Yuan, Qixiang Zhao, Hu Zhang, Hongyan Dong, Zhongming Zhang

**Affiliations:** ^1^ Department of Thoracic Cardiovascular Surgery Affiliated Hospital of Xuzhou Medical University Xuzhou China; ^2^ Morphological Research Experiment Center Xuzhou Medical University Xuzhou China; ^3^ Department of Cardiovascular and Thoracic Surgery Shanghai East Hospital Tongji University School of Medicine Shanghai China

**Keywords:** cardioprotection, mitophagy, Phosphorylation, pigment epithelium‐derived factor, protein kinase Cα, Unc‐51‐like kinase 1

## Abstract

Both decreased autophagy positive regulator AMP activated protein kinase (AMPK) level and promoted mitophagy are observed in oxygen‐glucose deprivation (OGD) cardiomyocytes treated with pigment epithelium‐derived factor (PEDF). This contradictory phenomenon and its underlying mechanisms have not been thoroughly elucidated. Our previous study reveals that PEDF increases the protein kinase Cα (PKCα) and phospho‐PKCα (p‐PKCα) contents to promote mitophagy. Thus, we investigated the association between PKCα and mitophagy. Here we identify an interaction between PKCα and Unc‐51‐like kinase 1 (ULK1), essential component of mitophagy. Further analyses show this is a direct interaction within a domain of ULK1 that termed the serine/threonine‐rich domain (S/T domain). Notably, a deletion mutant ULK1 that lacks the binding domain is defective in mediating PEDF‐induced mitophagy. Furthermore, we demonstrate that ULK1 is phosphorylated at Ser317/555/777 and Raptor is also phosphorylated by phospho‐PKCα. Phospho‐ULK1 (p‐ULK1) at these sites are all essential for PEDF‐induced mitophagy and reduce the release of mitochondrial ROS and DNA. This study therefore identifies a previously uncharacterized interaction between the ULK1 and PKCα that can replace the AMPK‐dependent mitophagy processes.

## 1 INTRODUCTION

Acute myocardial infarction (AMI), the primary component of cardiovascular disease, is the leading cause of morbidity and mortality worldwide.^1^, ^2^ Owing to the development of therapeutic intervention, morbidity and mortality have decreased markedly in recent decades. However, the burden of myocardial infarction and the incidence of heart failure as the end‐stage of left ventricular remodelling remain high.^3^

Mitochondria, the primary organelles of energy production and cell death, maintain their integrity by the intraorganellar proteolytic system and the dynamic nature of the mitochondrial population in ischemic cardiomyocytes. The severely damaged mitochondria that over‐produce reactive oxygen species are selectively degraded by an autophagic process, termed mitophagy, to reduce the oxidative burden and protect against apoptosis.^4^ Therefore, it is of great importance to mitochondrial quality control by mitophagy in cells, particularly, under different stresses such as ischemia, hypoxia, oxidative stress and DNA damage.^5^ AMP activated protein kinase (AMPK) is a heterotrimeric complex consisting of α‐, β‐ and γ‐ subunits that responds to energetic stress to maintain energy balance through phosphorylation of a diverse network of key metabolic pathways.^6^ Recently, AMP activated protein kinase (AMPK) has been reported as an activator of mitophagy that occurs through pleiotropic mechanism involving inhibition of the mammalian target of rapamycin (mTOR) complex and direct phosphorylation and activation of Unc‐51‐like kinase 1 (ULK1). Specifically, AMPK inhibits mTOR through phosphorylation of Raptor to induce14‐3‐3τ binding to Raptor.^7^ In addition, AMPK directly activates mitophagy through phosphorylation of ULK1 at several primary sites, while mTOR inhibits this process via ULK1 phosphorylation at Ser757.^8^

Pigment epithelium‐derived factor (PEDF) has gained attention as an endogenous secretory multifunctional protein, which is commonly expressed in various tissues^9^ and implicated in many cardiovascular diseases.^10^ Our pervious study indicated that PEDF expression is markedly decreased in oxygen‐glucose deprivation (OGD) cardiomyocytes and exogenous PEDF mediates the degradation of AMPK via an ubiquitin‐proteasome pathway.^11^ However, another study reveals that PEDF, surprisingly, promotes cardiomyocyte mitophagy via protein kinase Cα (PKCα)‐ULK1‐FUNDC1 pathway during OGD.^12^ These findings indicated that during OGD, PEDF‐induced ULK1 activation and mitophagy is probably dependent on PKCα rather than AMPK. It is noteworthy that the PKC family functions many signal transduction downstream,^13^ and PKCα is the predominant conventional PKC isoform expressed in mouse, rat and human heart.^14^ Furthermore, several studies have also demonstrated that mitophagy is associated with the PKC pathway.^15^, ^16^ Altogether, this inspired us to explore the physical relevance between PKCα and ULK1 in PEDF‐treated OGD cardiomyocytes.

In the current study, we investigated the importance of PKCα in controlling mitophagy. We find that during OGD, the substitution of AMPK with PKCα phosphorylates/activates ULK1 and Raptor to promote mitophagy. We also show that the phospho‐ULK1 induced by PEDF‐PKCα promotes FUNDC1‐mediated mitophagy and decrease the release of mitochondrial ROS and DNA, indicating that PEDF cardioprotective regulation of mitophagy is associated with PKCα rather than AMPK.

## 2 MATERIALS AND METHODS

Materials and expanded methods are presented in the Online Data S1.

### 2.1 Rat neonatal left ventricular cardiomyocyte isolation and culture

Neonatal cardiomyocytes were isolated from newborn Sprague‐Dawley (SD) rats. Cardiomyocytes were cultured in Dulbecco's modified Eagle's medium (Gibco) contain 4.5 g/L glucose with 10% foetal bovine serum and cultured at 37°C in a humidified atmosphere containing 5% CO_2_. Oxygen‐glucose deprivation (OGD) was achieved by culturing cells in glucose‐free DMEM (Gibco) without FBS supplement for glucose deprivation and in a tri‐gas incubator (Heal Force, Shanghai, China) saturated with 1% O_2_/5% CO_2_/94% N_2_ at 37°C for oxygen deprivation.

### 2.2 Cell viability and LDH release assay

Cell viability and LDH release were detected by Cell Counting Kit‐8 kit (CCK‐8; Dojindo, Tokyo, Japan) and LDH Cytotoxicity Assay Kit (Roche, Basel, Switzerland) according to the manufacturer's instructions.

### 2.3 Western blot

Protein samples from cardiomyocytes in the different groups were lysed for total protein extraction. Protein concentration was determined using a bicinchoninic acid (BCA) assay according to the manufacturer's protocol. Aliquots of equal amounts of protein from the lysate underwent Western blot analysis. The same membrane was stripped and reblotted with specific antibodies. For quantitative analysis, the bands were quantified using Image J software (NIH, Bethesda, USA) and the data were transformed and normalized relative to β‐actin, other than specifically noted, as the integral optical density (IOD) ratio.

### 2.4 In vitro kinase assay

ULK1 and Raptor were pre‐incubated with p‐PKCα in vitro. Cardiomyocytes were subjected to oxygen‐glucose deprivation for 4 hours and then immune precipitated with anti‐ULK1 and anti‐Raptor antibodies. The immune‐complex was incubated with 5 ng of recombinant rat PKC‐α proteins in kinase assay buffer containing 20 mmol/L HEPES at pH 7.4, 1 mmol/L EGTA, 0.4 mmol/L EDTA, 5 mmol/L MgCl2 and 0.05 mmol/L DTT (dithiothreitol) and supplemented with 0.2 mmol/L AMP and 0.1 mmol/L cold [γ‐^32^P]ATP (51963‐61‐2, PerkinElmer; Waltham, USA), for 15 minutes. The ULK1‐bound bead and Raptor‐bound bead were extensively washed with RIPA buffer (50 mmol/L Tris at pH 7.5, 150 mmol/L NaCl, 50 mmol/L NaF, 1 mmol/L EDTA, 1 mmol/L EGTA, 0.05% SDS, 1% Triton X‐100 and 0.5% deoxycholate), and kinase assay buffer and recovered by centrifugation. Immunoblotting was performed as previously described. Phosphorylations of p‐ULK1 and p‐Raptor proteins were determined by ^32^P‐ autoradiogram. Quantification of ^32^P‐Raptor and ^32^P‐ULK1 signal was performed using Phospho‐Imager and Kodak Multi Gage software.

### 2.5 Co‐immunoprecipitation

The whole proteins of cardiomyocytes were extracted using the special lysis buffer for immunoprecipitation. The lysates were centrifuged, and protein concentrations were measured by BCA assay. Part of the supernatant was used as input control, and the rest was immuneprecipated overnight at 4°C by gently rocking with anti‐ULK1 antibody. Approximately 4 μL antibodies were used for 400 μg total protein. Then, protein A/G agarose beads (Santa Cruz) was added to bind with the immunoprecipitates for 2 hours with gently shake at room temperature. Precipitated proteins were washed 3 times with lysis buffer and boiled with 5 × loading buffer, and immunoblotting was performed as previously described. Rabbit normal IgG (Santa Cruz) served as negative control.

### 2.6 Statistical analysis

Data are expressed as means ± SEM. Data between two groups were compared using two‐tailed Student's *t* test and multiple comparisons utilized one‐way ANOVA followed by Student–Newman–Keuls test. Statistical analysis was performed using PASW Statistic 21 (SPSS Inc., Chicago, USA). *P* < 0.05 was considered as significant difference.

## 3 RESULTS

### 3.1 PEDF promotes OGD cardiomyocyte mitophagy via PKCα‐ULK1‐FUNDC1 pathway

**Figure 1:**
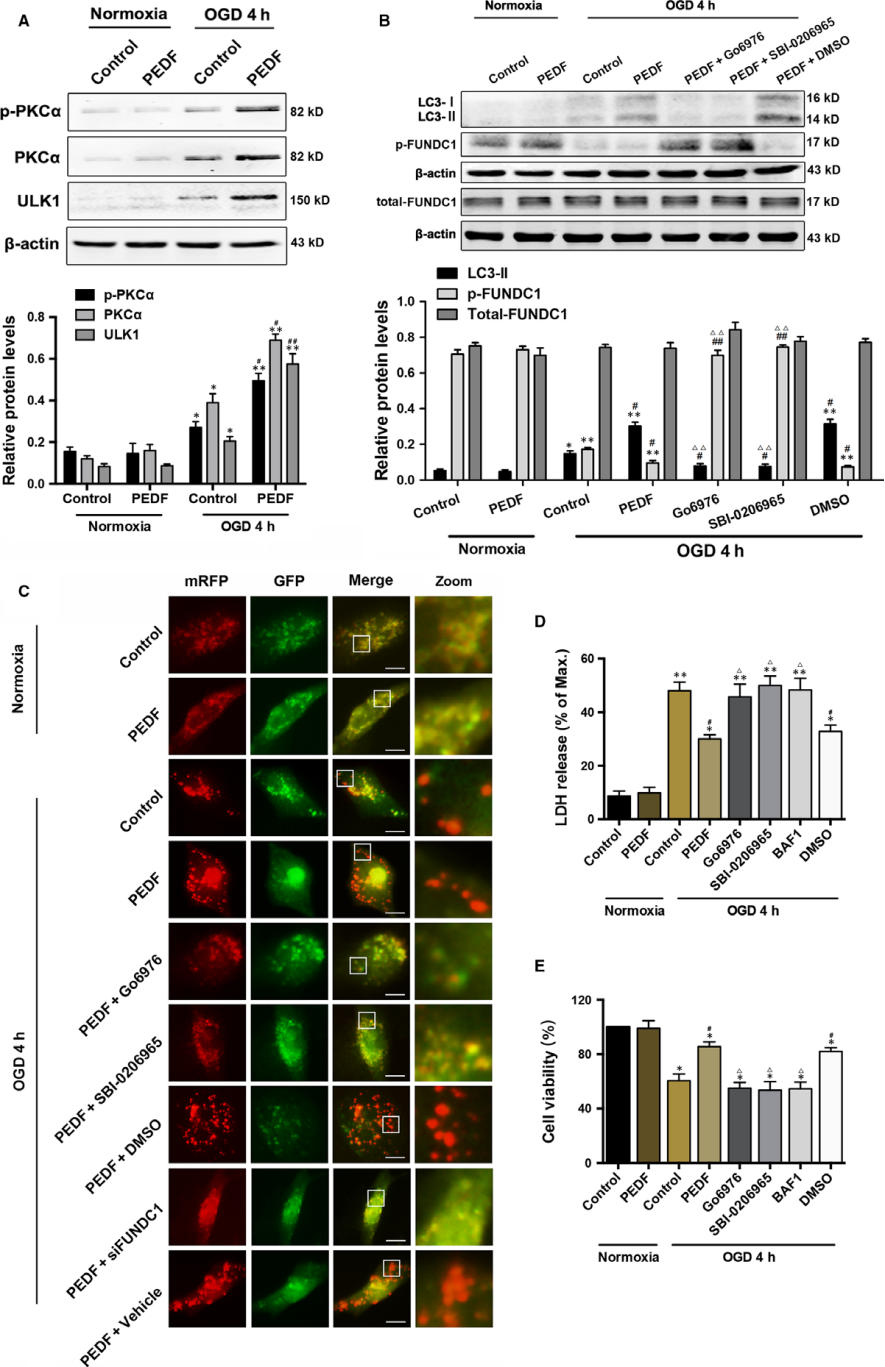
Exogenous PEDF in OGD cardiomyocytes is cardioprotective via PEDF‐PKCα‐ULK1‐FUNDC1 pathway. A, Protein levels of p‐PKCα, PKCα and ULK1 in cardiomyocytes treated with 10 nmol/L PEDF or not under normal conditions or subjected to oxygen‐glucose deprivation (OGD) for 4 hours, n = 6. B, Western bolt for LC3‐I, LC3‐II, p‐FUNDC1 and FUNDC1 in cardiomyocytes treated with PEDF (10 nmol/L), PKCα inhibitor Go6976 (1 μmol/L), ULK1 inhibitor SBI‐0206965(1 μmol/L) or DMSO before OGD 4 hours, n = 6. C, Representative images showing LC3 staining in different groups of cardiomyocytes infected with GFP‐RFP‐LC3 adenovirus for 24 hours, bar = 60 μm. Cardiomyocytes were treated with PEDF, Go6976, SBI‐0206965, DMSO and siFUNDC1 under normal conditions or before OGD for 4 hours, n = 30 from three independent experiments. D and E, Cell viability and cell death were assayed by (D) CCK‐8 and (E) LDH release assays. Cardiomyocytes were treated with PEDF, Go6976, SBI‐0206965, mitophagy inhibitor bafilomycin A1 (BAF1; 50 nmol/L) or DMSO under normal conditions or before OGD for 4 hours, n = 6. *P < 0.05, **P < 0.01 vs normal control, ^#^ *P* < 0.05, ^##^ *P* < 0.01 vsOGD control, ^Δ^ *P* < 0.05, ^ΔΔ^ *P* < 0.01 vs OGD+PEDF group

First, we examined the PEDF effects on OGD cardiomyocyte mitophagy. As shown in Figure 1A, p‐PKCα, PKCα and ULK1 levels were increased during oxygen‐glucose deprivation (OGD) compared with normoxia. PEDF could further significantly increase their content under OGD but not normal conditions. At mitochondria, FUNDC1 is an integral mitochondrial outer‐membrane protein. Structurally, FUNDC1 has two essential phosphorylation sites including Tyr18 and Ser13. Phosphorylated FUNDC1 generates steric hindrance for LC3II binding,^17^ thus effectively inhibiting mitophagy.^18^ Ischaemic or hypoxic stimulus alleviates FUNDC1 phosphorylation at Tyr18, leading to induction of mitophagy in ischaemia.^17^ And LC3‐I is lipidated to LC3‐II and associates to the cargo isolation membrane allowing for autophagosome formation.^19^ The expression of LC3‐II and phospho‐FUNDC1 (p‐FUNDC1) in PEDF‐treated OGD group was increased and decreased respectively compared with normal or OGD control group (Figure 1B). Pre‐treated with PKCα inhibitor Go6976 or ULK1 inhibitor SBI‐0206965 could abolish the effects of PEDF on LC3‐II and p‐FUNDC1. The expression of total‐FUNDC1 remained unchanged in all groups. Mitochondrial autophagy was detected by tandem GFP‐RFP‐LC3 adenovirus construct, which represents autophagosome formation as described.^20^ The rationale of this assay is based on the pH difference between the acidic autolysosome and the neutral autophagosome and the pH sensitivity differences exhibited by green fluorescent protein (GFP) and red fluorescent protein (RFP) to monitor progression from autophagosome to autolysosome. When an autophagosome fuses with a lysosome to form autolysosomes, the GFP moiety degrades from the tandem protein, but RFP‐LC3 maintains the puncta. As shown in Figure 1C, after infection with the GFP‐RFP‐LC3 adenovirus showing both fluorescent proteins. In addition to accumulation of LC3, there were more red punctum in PEDF‐treated OGD group than in normal or OGD control group, which was attenuated by Go6976, SBI‐0206965 or siFUNDC1. Mitochondrial autophagy was further detected by co‐localization of LC‐3 with Mito‐tracker Red‐stained mitochondria.^20^ As shown in Figure S1B, the intensity of LC‐3 that co‐localized with mitochondria was significantly increased in PEDF‐treated OGD group when compared to the normal or OGD control group, which was significantly attenuated by Go6976 or SBI‐0206965. Similarly, pre‐treated with Go6976 or SBI‐0206965 significantly suppressed PEDF‐induced increase of colocalization and fluorescence intensity of the Mito‐tracker Red (Figure S1C and D). Furthermore, cell viability inhibition and cell death of OGD cardiomyocytes were significantly decreased by PEDF, which reversed by Go6976 or SBI‐0206965 (Figure 1D,E). These results indicate that PEDF increase mitophagy of OGD cardiomyocytes through PEDF‐PKCα‐ULK1‐FUNDC1 axis.

### 3.2 PKCα interacts with ULK1 directly through the S/T domain

**Figure 2:**
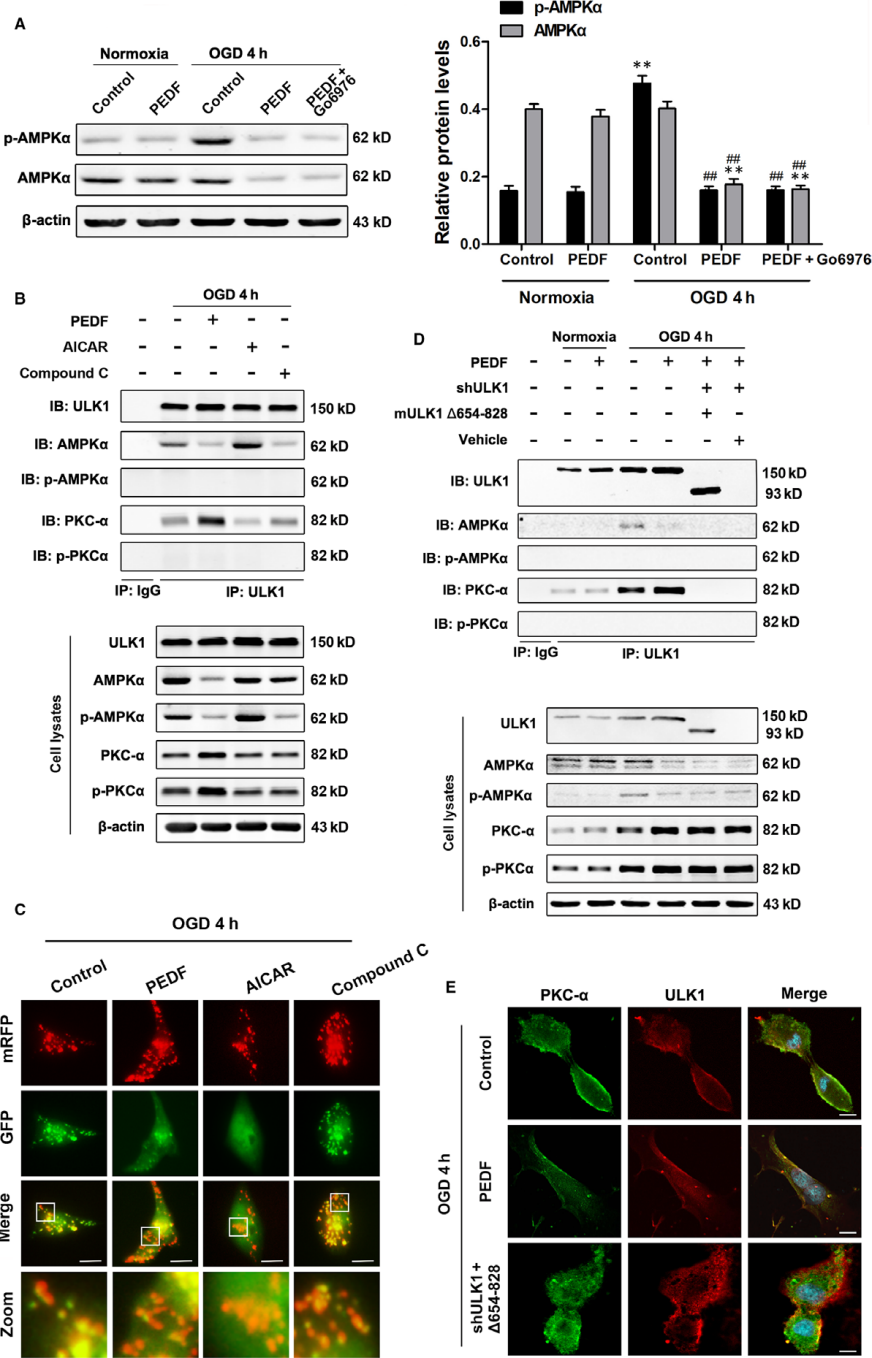
PKCα interacts with the S/T domain of ULK1. A, Protein levels of p‐AMPKα, AMPKα in cardiomyocytes treated with PEDF or not under normal conditions or subjected to OGD for 4 hours, n = 6. B, Cardiomyocytes were treated with PEDF, AMPKα inhibitor (Compound C, 10 μmol/L) or AMPKα activator (AICAR, 1 mmol/L), then immunoprecipitated with ULK1 antibody. ULK1, AMPKα, p‐AMPKα, PKCα and p‐PKCα were determined using their antibodies, n = 4. IP: immunoprecipitation, IB: immunoblotting. C, Representative images showing LC3 staining in different groups of Cardiomyocytes infected with GFP‐RFP‐LC3 adenovirus for 24 hours, bar = 60 μm. Cardiomyocytes were treated with PEDF, AICAR and Compound C before OGD for 4 hours, n = 30 from three independent experiments. D, Cardiomyocytes were treated with PEDF, lentiviral short hairpin RNA targeting rat ULK1 (shULK1), deletion mutant ULK1 (mULK1 Δ654‐828) or empty vehicle, then immunoprecipitated with ULK1 antibody. ULK1, AMPKα, p‐AMPKα, PKCα and p‐PKCα were determined using their antibodies, n = 4. IP: immunoprecipitation, IB: immunoblotting. E, Confocal immunofluorescence analysis of PKCα interaction with ULK1 in cardiomyocytes, bar = 60 μm. **P < 0.01 vs normal control, ^##^ *P* < 0.01 vs OGD control

It is well established that AMPKα participates in ULK1‐mediated mitophagy, so we determined the expression of AMPKα. Similar to our previous study, phospho‐AMPKα (p‐AMPKα) was increased during OGD for 4 hours and both p‐AMPKα and AMPKα levels were significantly decreased to ~50% with the treatment of PEDF compared with OGD control, and Go6876 has no effect on the expressions of AMPKα and p‐AMPKα with PEDF (Figure 2A) or not (Figure S2) when compared to OGD control group or PEDF‐treated OGD group, respectively. These findings reveal that PEDF‐promoted mitophagy in OGD cardiomyocytes was probably independent with AMPK. Previous study has reported that AMPK binds to the serine/threonine‐rich domain (S/T domain) of ULK1 which is required for ULK1‐mediated autophagy.^21^ Thus, it is of interest to determine whether PKCα could directly interact with S/T domain of ULK1. As shown in Figure 2B,C, under OGD conditions, ULK1 is combined with part of AMPKα and PKCα. Pre‐treated with PEDF and AICAR (AMPK activator) significantly increased the combination of PKCα and AMPKα with ULK1, respectively. Importantly, AMPK inhibitor Compound C significantly reduced the combination of AMPKα with ULK1 and increased slightly the binding of PKCα to ULK1. Furthermore, both PEDF and AICAR significantly increased the cardiomyocyte mitophagy, and Compound C had no significant effect on mitophagy compared with OGD group. These resulus demonstrated that simple inhibition of AMPK did not reduce ULK1 activation and mitophagy, the compensatory increase in PKC maintained ULK1 and mitophagy activity. These also further demonstrate that PKC can activate ULK in place of AMPK to promote mitochondrial autophagy. To confirm the relationship between PKCα and ULK1, ULK1 was pulled down by immunoprecipitation with a specific antibody which demonstrated that PKCα was bound to ULK1 in the presence of PEDF during OGD (Figure 2D). Then we generated a deletion mutant ULK1 (Δ654‐828) that lacks the AMPK binding domain. Notably, this deletion mutant ULK1 could not bind to PKCα. Similar results were observed by confocal laser scanning microscope which reinforce the notion that PKCα binding to ULK1 through the S/T domain with PEDF treatment under OGD conditions (Figure 2E).

### 3.3 PKCα is required for the phosphorylation of Raptor and ULK1 at Ser317, 555 and 777

**Figure 3:**
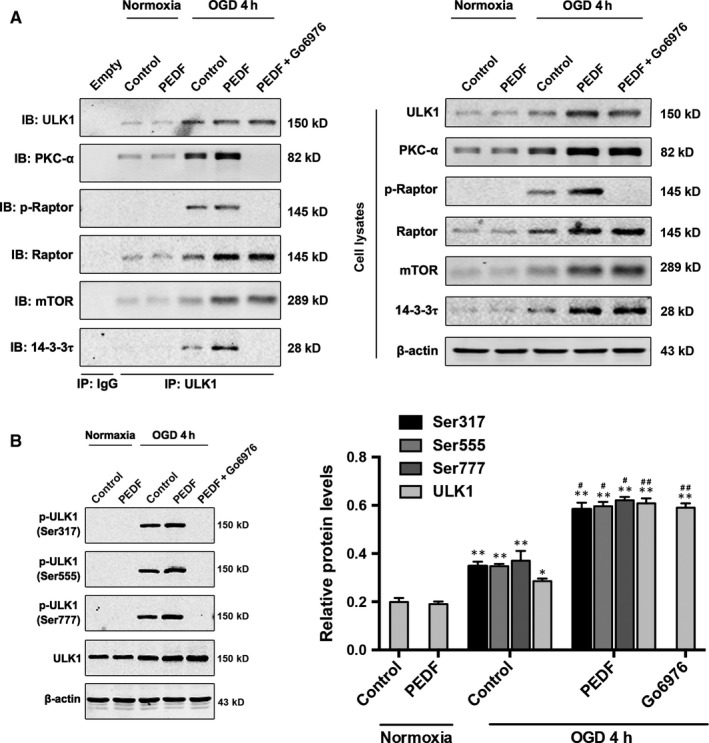
PKCα induces the phosphorylation of Raptor and ULK1 at S317, S555 and S777**.** A, Cardiomyocytes were treated with PEDF or PEDF+Go6976 and then immunoprecipitated with ULK1 antibody. ULK1, PKCα, p‐Raptor, Raptor, mTOR and 14‐3‐3τ were determined using their antibodies, n = 5. (B) Western bolt for p‐ULK1 (Ser317, 555, 777) and ULK1 in cardiomyocytes treated with PEDF or PEDF+Go6976 under normal conditions or before OGD 4 hours, n = 6. *P < 0.05, **P < 0.01 vs relative normal control, ^#^ *P* < 0.05, ^##^ *P* < 0.01 vs relative OGD control

Next, we determine whether PKCα could phosphorylate ULK1 and Raptor.ULK1 was pulled down with anti‐ULK1 antibody and results demonstrated that PKCα interacted with the ULK1‐Raptor‐mTOR complex and Raptor was phosphorylated to recruit 14‐3‐3τ with the treatment of PEDF during OGD (Figure 3A). PKCα inhibitor Go6976 could significantly decrease the binding of PKCα and ULK1, thus reduced the phosphorylation of Raptor and the recruitment of 14‐3‐3τ. Furthermore, it is reported that AMPK promotes autophagy by directly activating ULK1 through phosphorylation of Ser555,^22^ Ser317 and Ser777.^8^ Hence, we aimed to verify whether PKCα phosphorylated ULK1 through these sites. As shown in Figure 3B, impressively, the phosphorylation of ULK1 at Ser555, Ser317 and Ser777 was significantly increased in PEDF‐treated OGD cardiomyocytes compared with normal or OGD control. Pre‐treated with Go6976 abolished the increase of p‐ULK1 by PEDG. These results indicated that both phosphorylation of Raptor and ULK1 and recruitment of 14‐3‐3τ were related to PEDF‐promoted PKCα level.

### 3.4 The phosphate group of phospho‐ULK1 (p‐ULK1) and phospho‐Raptor (p‐Raptor) is from phospho‐PKCα

**Figure 4:**
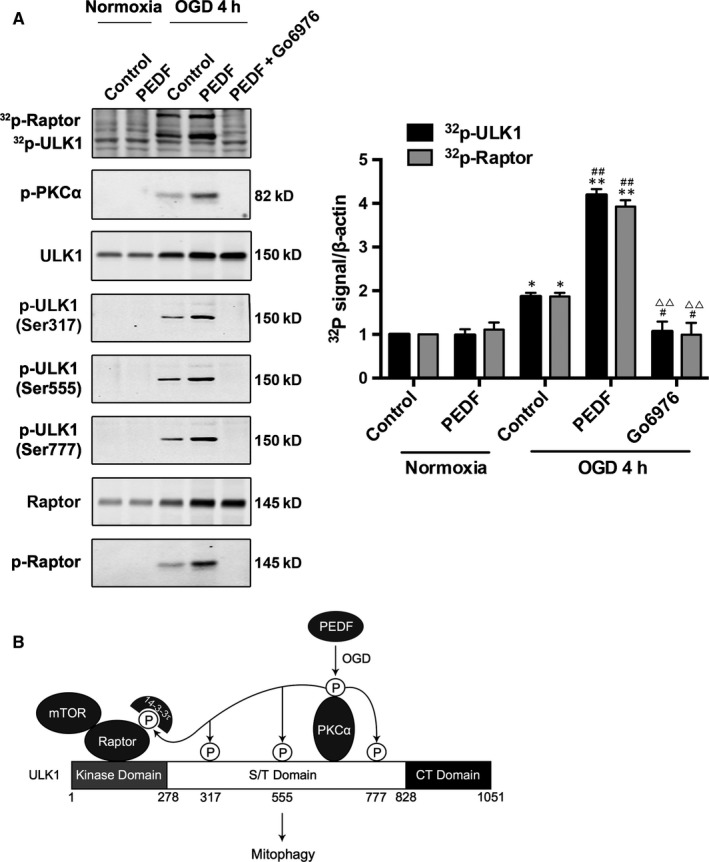
Phospho‐PKCα contributes its phosphate group to phospho‐ULK1 and phospho‐Raptor. A, Cardiomyocytes were deprived of oxygen and glucose 4 hours as indicated after treated with PEDF and PEDF+Go6976. p‐ULK1 and p‐Raptor were immunoblotted and phosphorylation was examined by ^32^P‐autoradiogram. Proteins were resolved by SDS‐PAGE and visualized with autoradiogram (top) and Western blot (WB; bottom), n = 6. B, Schematic representation of PEDF‐mediated p‐PKCα induces the phosphorylation of ULK1 and suppresses the inhibitory effect of mTOR on the ULK1 autophagic complex. *P < 0.05, **P < 0.01 vs normal control, ^#^ *P* < .05, ^##^ *P* < 0.01 vs OGD control, ^ΔΔ^ *P* < 0.01 vs OGD+PEDF group

Previous findings indicated that phospho‐PKCα (p‐PKCα) was also increased in PEDF‐treated cardiomyocytes (Figure 1A). Therefore, it is of interest to determine whether the phosphate group of p‐ULK1 and p‐Raptor were transferred from p‐PKCα. We labelled ^32^p‐PKCαto incubate with ULK1 and Raptor and the phosphorylation of p‐ULK1 and p‐Raptor were examined by ^32^P‐autoradiogram (Figure 4A). Our results reveal that both p‐ULK1 and p‐Raptor were labelled with ^32^P suggesting that the phosphate group in p‐ULK1 and p‐Raptor were derived from p‐PKCα. PKCα inhibitor Go6976 significantly suppressed PKCα‐induced phosphorylation of ULK1 (Ser317, 555 and 777) and Raptor. In addition, the level of p‐PKCα was also decreased by Go6976 treatment.

### 3.5 PEDF steered phospho‐ULK1 is important for FUNDC1‐mediated mitophagy

**Figure 5:**
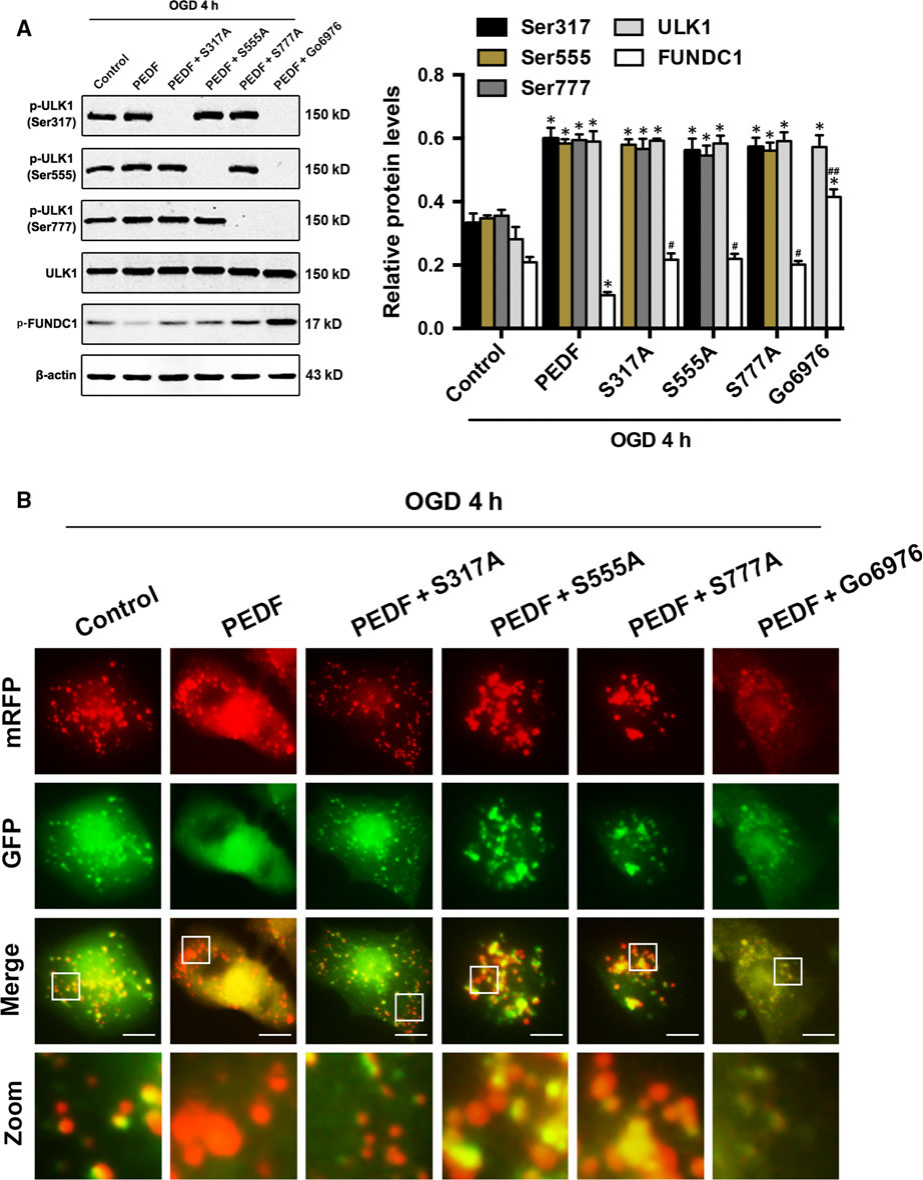
Phospho‐ULK1 induced by PEDF is important for FUNDC1‐mediated mitophagy. A, Western bolt for p‐ULK1 (Ser317, 555, 777), ULK1 and p‐FUNDC1 in cardiomyocytes treated with PEDF or PEDF+Go6976 before OGD 4 for hours, n = 6. S317/555/777A mutants were transfected into cardiomyocytes as indicated. *P < 0.05 vs relative normal control, ^#^ *P* < 0.05, ^##^ *P* < 0.01 vs relative OGD control. (B) Representative images showing LC3 staining in different groups of cardiomyocytes infected with GFP‐RFP‐LC3 adenovirus for 24 hours, bar = 60 μm. Cardiomyocytes were treated with PEDF or PEDF+Go6976before OGD 4 hours, n = 30 from three independent experiments

Next, the functional significance of PEDF‐induced Ser317, Ser555 and Ser777 phosphorylation in ULK1 activation was examined. PEDF markedly activated wild‐type ULK1, but did not activate the phosphorylation defective S317A, S555A and S777A mutant (Figure 5A). The expression of p‐FUNDC1 in phosphorylation defective ULK1 mutant groups was significantly increased and decreased compared with PEDF group and PEDF+Go6976 group, respectively. Mitochondrial autophagy was further detected by tandem GFP‐RFP‐LC3 adenovirus construct. As shown in Figure 5B, there were less red puncta in phosphorylation defective ULK1 mutant groups than PEDF group and pre‐treated with Go6976 further reduced the quantitative of the red puncta. Similar results were observed in mitochondrial autophagy detected by co‐localization of LC‐3 with Mito‐tracker Red‐stained mitochondria. As shown in Figure S1E, the intensity of LC‐3 that co‐localized with mitochondria in phosphorylation defective ULK1 mutant groups was markedly increased and decreased compared with PEDF group and PEDF+Go6976 group, respectively. Consistently, phosphorylation defective mutants significantly attenuated PEDF‐induced increase in colocalization and fluorescence intensity of the Mito‐tracker Red (Figure S1F and G). Pre‐treated with Go6976 further reduced the colocalization and fluorescence intensity. These findings demonstrated that the phosphorylation of ULK1 at Ser317, 555 and 777 induced by PEDF was all essential for FUNDC1‐mediated mitophagy.

### 3.6 PEDF reduces the release of mitochondrial ROS and DNA into cytosol

**Figure 6:**
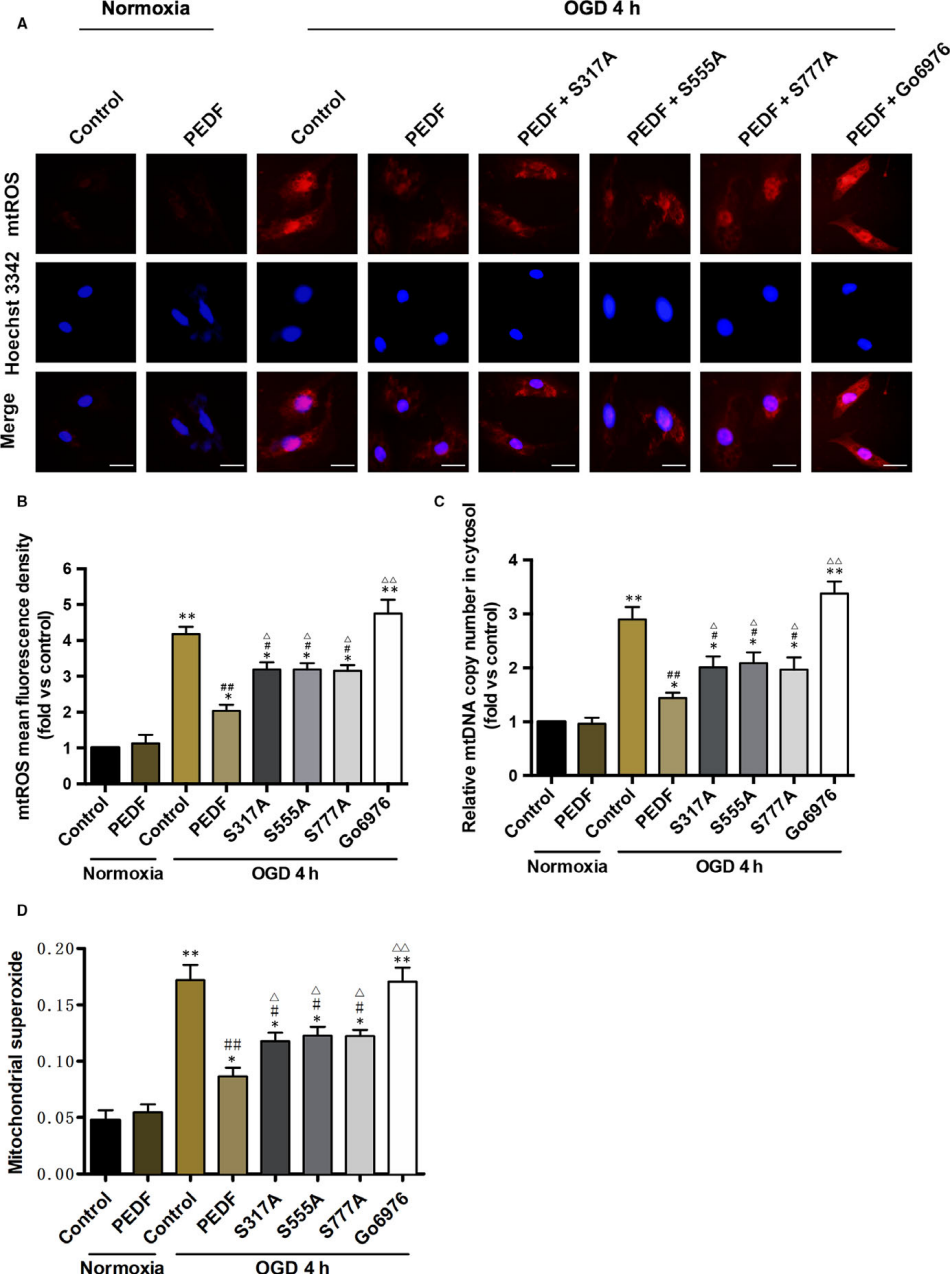
The release of mitochondrial ROS and DNA in cytosol is decreased by PEDF‐induced phospho‐ULK1. A and B, Fluorescence microscope of (A) Mito‐SOX Red‐labelled ROS production and release in cytosol and quantification of (B) fluorescence density, bar = 100 μm, n = 30 from 3 independent experiments. Cardiomyocytes were treated with PEDF or PEDF+Go6976 under normal conditions or before OGD 4 hours. S317/555/777A mutants were transfected into cardiomyocytes as indicated. C, Cytosolic mitochondrial DNA copy number was measured by quantitative PCR, n = 7. Wild type or S317/555/777A mutational cardiomyocytes were treated with PEDF or Go6976 under normal conditions or before OGD 4 hours. D, Quantification of mitochondrial superoxide was detected with flow cytometric, n = 3. Wild type or S317/555/777A mutational cardiomyocytes were treated with PEDF or Go6976 under normal conditions or before OGD 4 hours. *P < 0.05, **P < 0.01 vs normal control, ^#^ *P* < 0.05, ^##^ *P* < 0.01 vs OGD control, ^Δ^ *P *< 0.05, ^ΔΔ^ *P* < 0.01 vs OGD+PEDF group

Previous studies reveal that the major source of mitochondrial damage comes from imbalanced production of reactive oxygen species (mtROS), and mtROS subsequently impair mitochondrial structure and mitochondrial biogenesis via oxidative modifications on macromolecules, such as mitochondrial DNA (mtDNA).^23^, ^24^ Thus, we next evaluated the levels of mtROS and mtDNA. Phosphorylation defective ULK1 mutant at Ser317, 555 and 777 were all significantly decreased, although not completely, the PEDF‐induced reduction in mtROS and mtDNA content compared with OGD group (Figure 6B,C). As expected, Go6976 could drastically abolish the decrease in mtROS and mtDNA by PEDF. In addition, similar results were observed in mitochondrial superoxide detected by flow cytology. Quantitative of mitochondrial superoxide significantly reduced in PEDF‐treated OGD group compared with OGD control group, and Go6976 could drastically abolish the decrease of mitochondrial superoxide by PEDF. Phosphorylation defective ULK1 mutant at Ser317, 555 and 777 was all significantly decreased, although not completely, the PEDF‐induced reduction in mitochondrial superoxide compared with OGD group (Figure 6D). Together, our data demonstrated the importance of Ser317, Ser555 and Ser777 phosphorylation of PEDF‐induced and PKCα‐dependent ULK1 activation in mitigating mitochondrial damage.

## 4 DISCUSSION

In mammalian cells, PKCα consists of 672 amino acids and is distributed in all tissues, in contrast to other PKC isotypes whose expression is restricted in the particular tissues.^25^, ^26^ PKCα is activated by a variety of stimuli, including signals binding to guanine nucleotide binding protein‐coupled receptors and to tyrosine kinase receptors,^25^, ^26^, ^27^, ^28^, ^29^ and also physical stresses like hypoxia.^30^ The kinase activity of PKCα is regulated by phosphorylation of conserved residues in its kinase domain: the activation‐loop site Thr497, the autophosphorylation site Thr638 and the hydrophobic C‐terminal site Ser657.^31^, ^32^ PKCα exhibits almost no activity without phosphorylation at these sites, and phosphorylation of Ser657 is currently used as an indicator for PKCα activation.^31^, ^33^

In the present study, we first presented a physical relevance between PKCα and ULK1, which phosphorylates ULK1 at Ser317, 555, 777 and Raptor to promote mitophagy in cardiomyocyte treated with PEDF (Figure 4B). It is known that AMPK, a classical ULK1‐binding partner, binds to amino acids 654‐828 within the S/T domain of ULK1.^21^ However, in our study, the ischaemic cardiomyocytes treating with exogenous PEDF presented a decrease in AMPK and increase in PKCα. Alternatively, PKCα occupied the AMPK binding‐domain subsequently. Furthermore, our results indicated an important mechanism that p‐PKCα (Ser 657) binds to and phosphorylates ULK1 and Raptor in PEDF‐treated OGD cardiomyocytes rather than PKCα. As PKCα inhibitor Go6976 decrease both the PKCα and p‐PKCα level, we hypothesized that Go6976 disturbed the interaction between PKCα and ULK1 is possibly associated with a decrease in p‐PKCα level and PKCα activation.

As an autophagy‐initiating kinase, the mechanism of ULK1 regulation is central to understanding mitophagy regulation. This study demonstrated a novel biochemical mechanism of ULK1 activation by upstream signals and the functional importance of this regulation in autophagy induction. Under oxygen‐glucose deprivation, the activated PKCα inhibits mTOR to lead ULK1‐PKCα interaction. Activated‐PKCα then phosphorylates ULK1 on Ser 317, 555 and 777 activates ULK1 kinase and eventually leads to mitophagy induction. The coordinated phosphorylation of ULK1 by p‐PKCα and mTOR may provide a mechanism for signal integration and, thus cells can properly respond to the complex extracellular milieu. Although PKCα may phosphorylate additional sites that may contribute to ULK1 activation, phosphorylation of Ser 317/Ser 555/Ser 777 is required for ULK1 activation and efficient mitophagy induction in response to oxygen‐glucose deprivation. As PEDF does not promote cardiomyocyte p‐PKCα level and mitophagy under normoxic conditions, oxygen‐glucose deprivation may be one of the necessary conditions for PEDF to play a cardioprotective role. But the specific mechanism remains to be further investigation.

Taken together, our data reveal a novel mechanism that PEDF‐induced p‐PKCα replaces AMPK‐ULK1 interaction and phosphorylates ULK1 at Ser 317, 555 and 777 to promote mitophagy. Tactic for targeting of PEDF mitophagy pathways in AMI may be of therapeutic benefit suggesting a new strategy to protect cell viability and fight cardiovascular diseases.

## CONFLICTS OF INTEREST

The authors confirm that there are no conflicts of interest.
