# Supplementary material for: PKCα replaces AMPK to regulate mitophagy: Another PEDF role on ischaemic cardioprotection
Source: J Cell Mol Med. 2018 Sep 19;22(11):5732–42. doi: 10.1111/jcmm.13849 (PMC6201373; doi:10.1111/jcmm.13849)
Supplement: Supplementary file 1 [file JCMM-22-5732-s001.docx]

**Supplemental Data**

Supplemental Methods

Supplemental References

Supplemental Figure Legend

**Supplemental Methods**

**Materials.** Antibodies against PKC-α (2056), ULK1(8054), LC3-I/ II (4108), LC3-II (2775), AMPKα (5832), p-AMPKα (Thr^172^, 2535), p-Raptor (Ser^792^, 2083), Raptor (2280), mTOR (2983), 14-3-3τ (9638), p-ULK1 (Ser^317^, 12753) and p-ULK1 (Ser^555^, 5869) were obtained from Cell Signaling Technology (CST, Beverly, USA). Anti-pULK1 (Ser^777^) antibody was prepared by immunizing rabbits (Biomyx, San Diego, USA). Antibody for p-PKCα (Ser^657^, 07-790) was obtained from Millipore (Darmstadt, Germany). Antibody of β-actin (66009-1-lg) was purchased from Proteintech (Rosemont, USA). Antibody for FUNDC1 (ab74834) was bought from Abcam (Cambridge, UK). Anti-FUNDC1 (p-Tyr18) polyclonal antibody generated by immunizing a rabbit with purified FUNDC1 phospho-peptides followed by affinity purification (Abgent, San Diego, USA). Antibody for α-sarcomeric actin (α-SCA, SAB4200689) was bought from Sigma-Aldrich (Darmstadt, Germany). Go6976 (S7119), SBI-0206965 (S7885), Bafilomycin A1 (BAF1; S1413), Compound C (S7306, 10μmol/L) and AICAR (S1802, 1mmol/L) were purchased from Selleck Chemicals (Houston, USA). Mitochondrial superoxide (Mito-SOX) indicator (M36008) was purchased from Thermo Fisher Scientific (Waltham, USA). Hoechst 3342 was purchased from Keygen Biotech (Nanjing, China). Mito-Tracker Red was purchased from Invitrogen (Carlsbad, USA). The Autophagy Indicator pHBmTur-Mito (HB-AP210 0001) was purchased from Han Heng Biological Technology Co., Ltd. (Shanghai, China). Recombinant rat PEDF (GenBankTM Accession Number NM_177927) was synthesized by Cusabio Biotech (Wuhan, China).

**Rat ventricular cardiomyocyte** **isolation, culture and transfection.** Neonatal cardiomyocytes were isolated from 1-day-old newborn SD rats [1]. Briefly, neonatal rats were anesthetized with sodium pentobarbital and implement decapitation. Hearts were rapidly removed into dishes on ice, and discarded the vessels and atria. Then ventricles were dissected and minced into 1 mm^3^ pieces and transferred to a sterile tube, washed in cold PBS solution (136.9 mmol/L NaCl, 2.7 mmol/L KCl, 8.1 mmol/L Na_2_HPO_4_, 1.5 mmol/L KH_2_PO_4_, pH 7.3) to remove blood clots. Minced tissue was digested in a PBS solution supplemented with 1 mg/ml trypsin, 1 mg/ml collagenase type II, and 0.2 mg/ml glucose for 5 minutes at 37°C and incubated with 0.1 mmol/L BrdU to selectively enrich for cardiomyocytesby inhibiting the growth of cardiac fibroblasts. Cardiomyocytes were then purified using differential adhesion method and confirmed by immunofluorescence of α-SCA (Figure S1). Cardiomyocytes were plated on 60 mm dishes (Corning, New York, USA) at a density of 25,000 cells/cm^2^ in Dulbecco’s modified Eagle medium (DMEM, Gibco, Waltham, USA) containing 4.5 g/L glucose and supplemented with 10% fetal bovine serum (FBS, Gibco) and 1% Penicillin-Streptomycin solution (Invitrogen), cultured at 37°C in a humidified atmosphere containing 5% CO_2_. Oxygen-glucose deprivation (OGD) was achieved by culturing cells in glucose-free DMEM (Gibco) without FBS supplement for glucose deprivation and in a tri-gas incubator (Heal Force, Shanghai, China) saturated with 1% O_2_/5% CO_2_/94% N_2_ at 37°C for oxygen deprivation for the indicated time periods. The lentiviral short hairpin RNA (shRNA) targeting rat ULK1 were purchased from Open Biosystems (Lafayette, USA). For interfere the ULK1 expression, shULK1 were transfected following the manufacturer’s protocol. After 8 hours, transfection medium was removed and fresh medium was added. For transient expression of proteins, cardiomyocytes were transfected with the indicated mutant Ulk1 (mUlk1) recombinant DNA plasmidsusing Lipofectamine 2000 (Invitrogen) following manufacturer’s protocol. Cells were harvested 48 hours after transfection for co-immunoprecipitation assay or western blot analysis.

**Cell viability and** **LDH release****assay.** Cardiomyocytes were seeded in 96-well plates at a concentration of 1x10^4^/mL. After treatment, cell viability was detected by using a Cell Counting Kit-8 kit (CCK-8; Dojindo, Tokyo, Japan). Absorbance at 450 nm was measured with a microplate reader (BioTek Synergy 2, Vermont, USA). The means of the optical density (OD) measurements from 6 wells of the indicated groups were used to calculate the percentage of cell viability. The LDH activity in cardiomyocytes released into medium was assessed using an LDH Cytotoxicity Assay Kit (Roche, Basel, Switzerland) according to the manufacturer's instructions.

**siRNA and Mutant plasmid.** The FUNDC1 scramble RNA interference sequence was: sense 5’-UUCUCCGAACGUGUCACGUTT-3’; antisense 5’-ACGUGACACGUUCGGAGAATT -3’. RNA oligonucleotides were transfected into cells using Lipofectamine 2000 (Invitrogen) according to the manufacturer’s protocol. The cDNA encoding rat ULK1 was cut off by HindIII from the plasmid CMV-ULK1-WT (Addgene; Cambridge, USA) and was cloned into the expression vector p3XFlag-myc-CMV-24. Mutagenesis was performed using a Quik Change Site-Directed Mutagenesis Kit (Stratagene, Santa Clara, USA). Serine 317, 555 and 777 were mutated to alanine (S317A, S555A and S777A, respectively). The following primers were used: 5'-CCTGGCATCTCCCCCGGCCCTGGGGGAGATGCC-3' (mutant ULK1 S317A forward), 5'-GGCATCTCCCCCAGGGCCGGGGGAGATGCCAGG-3' (S317A reverse); 5'-GGGCTGCCGCCTGCACGCTGCCCCCAACCTTTCG-3' (S555A forward), 5'-CGAAAGGTTGGGGGCAGCGTGCAGGCGGCAGCCC-3' (S555A reverse); 5'-GAATGTTCTCAGTGGGCGCTTCCAGCTCCCTGGGC-3' (S777A forward), 5'-GCCCAGGGAGCTGGAAGCGCCCACTGAGAACATTC-3' (S777A reverse). The mutant plasmids were sequenced and the mutations were confirmed.

**Immunofluorescence.** Cardiomyocytes were ﬁxed for 15 minutes with 4% paraformal dehyde, blocked with BSA and incubated with primary antibodies and subsequently with secondary antibodies (Life Technologies, Waltham, USA). After final washing, the cover slips were mounted on slides using 50% glycerin. Then the cells were observed using a fluorescence microscope (Olympus, Tokyo, Japan) or confocal laser scanning microscope (Olympus).The co-localization rates and intensity of LC3/Mito-tracker Red were analyzed by Image-Pro Plus (Media Cybernetics, Silver Spring, USA).

To monitor the autophagy, the tandem GFP-RFP-LC3 adenovirus construct obtained from Hanbio Inc (Shnaghai, China) was used in this study. This tandem GFP-RFP-LC3 construct capitalizes on the pH difference between the acidic autolysosome and the neutral autophagosome and the pH sensitivity differences exhibited by GFP (green fluorescent protein) and RFP (red fluorescent protein) to monitor progression from the autophagosome to autolysosome. In brief, to perform image-based analysis of autophagy, neonatal cardiomyocytes were infected with tandem GFP-RFP-LC3 adenovirus for 2 hours and then were cultured with normal medium for 24 hours, and then the cells were treated and imaged for GFP and RFP by using ﬂuorescence microscopy.

**Western blot**. For whole cell lysates, cells were lysed with Cell Total Protein Extraction Kit (Sangon Biotech, Shanghai, China). Protein concentrations were measured using a bicinchoninic acid (BCA) assay. Equivalent amount of protein was prepared and separated by 8-12% sodium dodecyl sulfate-polyacrylamide gel (SDS-PAGE) and electro-transferred to nitrocellulose membranes (Millipore). Then probed with indicated antibody 4°C overnight, and incubated with secondary antibody for 2 hours at room temperature. Finally, signals were detected by Odyssey Infrared Imaging System (Li-Cor Biosciences, Lincoln, USA). Digitized images were analyzed using Image J (NIH, Bethesda, USA). For all western blot analyses, other than specifically noted, protein levels were calculated from the ratio of corresponding protein/β-actin.

**In Vitro Kinase Assay.** To evaluate the effects of phosphorylation on ULK1 and Raptor kinase activity, ULK1 and Raptor were pre-incubated with p-PKCα in vitro. First, cardiomyocytes were deprived of oxygen and glucose for 4 hours and the ULK1 and Raptor were immunoprecipitated with anti-ULK1 and anti-Raptor antibodies. Then the immune-complex was incubated with 5ng of recombinant rat PKC-α proteins in kinase assay buffer containing 20 mmol/L HEPES at pH 7.4, 1 mmol/L EGTA, 0.4 mmol/L EDTA, 5 mmol/L MgCl2 and 0.05 mmol/L DTT (dithiothreitol) and supplemented with 0.2 mmol/L AMP and 0.1 mmol/L cold [γ-^32^P] ATP (51963-61-2, PerkinElmer; Waltham, USA), for 15 min. The ULK1-bound bead and Raptor-bound bead were extensively washed with RIPA buffer (50 mmol/L Tris at pH 7.5, 150 mmol/L NaCl, 50 mmol/L NaF, 1 mmol/L EDTA, 1 mmol/L EGTA, 0.05% SDS, 1% Triton X-100 and 0.5% deoxycholate) and kinase assay buffer and recovered by centrifugation. Immunoblotting was performed as previously described. Phosphorylations of p-ULK1 and p-Raptor proteins were determined by ^32^P- autoradiogram. Quantification of ^32^P-Raptor and ^32^P-ULK1 signal was done using Phospho-Imager and Kodak Multi Gage software.

**Co-immunoprecipitation.** The whole proteins of cardiomyocytes were extracted using the special lysis buffer for immunoprecipitation. The lysates were centrifuged and protein concentrations were measured by BCA assay. Part of the supernatant was used as input control and the rest was immunoprecipated overnight at 4ºC by gently rocking with anti-ULK1 antibody. Approximately 4 μL antibodies were used for 400 μg total protein. Then protein A/G agarose beads (Santa Cruz) was added to bind with the immunoprecipitates for 2 hours with gently shake at room temperature. Precipitated proteins were washed 3 times with lysis buffer and boiled with 5× loading buffer, and immunoblotting was performed as previously described. Rabbit normal IgG (Santa Cruz) served as negative control.

**Determination of mito-SOX by flow cytometry.** The Mito-SOX indicator was used for the following assay. Apply 3 mL of 5 μM Mito-SOX reagent working solution to cover neonatal cardiomyocytes. Incubate cells for 10 minutes at 37˚C, protected from light. Neonatal cardiomyocytes (1x10^6^ per group) were collected, washed three times with warm buffer, resuspended with 500 µl Hankʼs balanced salt solution with calcium and magnesium, Then, the cells were analyzed using a ﬂow cytometer (BD Biosciences, Franklin Lakes, NJ, USA).

**Mitochondrial ROS production assay.** Cardiomyocytes were seeded into a 48-well plate and subsequently loaded with 200 μL Mito-SOX for 10 minutes. After 3 washing steps with PBS, nuclei were counterstained with Hoechst 3342 stain for 15 minutes. Then the cells were observed using a fluorescence microscope (Olympus, Tokyo, Japan) and the mtROS mean density were analyzed by Image-Pro Plus (Media Cybernetics).

**Measurement of mtDNA in Cytosol by qPCR.** The total DNA of cardiomyocytes was extracted using a DNeasy Blood & Tissue Kit (QIAGEN, Dusseldorf, Germany). The mtDNA in cytosol was extracted further by using a multiple centrifugation as described [2]. The mtDNA copy number was then measured by real-time polymerase chain reaction (qPCR) using Light Cycler 480II (Roche, Basel, Switzerland) with SYBR Green PCR Master Mix (Applied Biosystems, Waltham, USA). To determine the relative mitochondrial DNA level, the mtDNA copy number was normalized to nuclear DNA levels in a ratio of cytochrome c oxidase 1 (mtCO1) DNA vs. nuclear DNA (encoding 18S ribosomal RNA) [3, 4]. The sequences of the primer are as follows: 5'-CAGCCGTCCTACTACTTCTCTCA-3' (mtCO1 forward), 5'-GATTGGGTCTCCACCTCCA-3' (mtCO1 reverse); 5'-GACTCAACACGGGAAACCTC-3' (18S forward), 5'-AGACAAATCGCTCCACCAAC-3' (18S reverse).

**Statistical analysis.** Data are expressed as means ± SEM. Data between two groups were compared using two-tailed Student’s t test and multiple comparisons utilized one-way ANOVA followed by Student-Newman-Keuls test. Statistical analysis was performed using PASW Statistic 21 (SPSS Inc., Chicago, USA). P<0.05 was considered as significant difference.

**Supplemental References**

1. Sun M, Ouzounian M, de Couto G, et al. Cathepsin-L ameliorates cardiac hypertrophy through activation of the autophagy-lysosomal dependent protein processing pathways. J Am Heart Assoc. 2013 Apr 22;2(2):e000191. doi: 10.1161/JAHA.113.000191. PubMed PMID: 23608608; PubMed Central PMCID: PMCPMC3647266.

2. Nakahira K, Haspel JA, Rathinam VA, et al. Autophagy proteins regulate innate immune responses by inhibiting the release of mitochondrial DNA mediated by the NALP3 inflammasome. Nat Immunol. 2011 Mar;12(3):222-30. doi: 10.1038/ni.1980. PubMed PMID: 21151103; PubMed Central PMCID: PMCPMC3079381.

3. Tal MC, Sasai M, Lee HK, et al. Absence of autophagy results in reactive oxygen species-dependent amplification of RLR signaling. Proc Natl Acad Sci U S A. 2009 Feb 24;106(8):2770-5. doi: 10.1073/pnas.0807694106. PubMed PMID: 19196953; PubMed Central PMCID: PMCPMC2650341.

4. Lemasters JJ, Theruvath TP, Zhong Z, et al. Mitochondrial calcium and the permeability transition in cell death. Biochim Biophys Acta. 2009 Nov;1787(11):1395-401. doi: 10.1016/j.bbabio.2009.06.009. PubMed PMID: 19576166; PubMed Central PMCID: PMCPMC2730424.

**Supplemental Figure Legend**


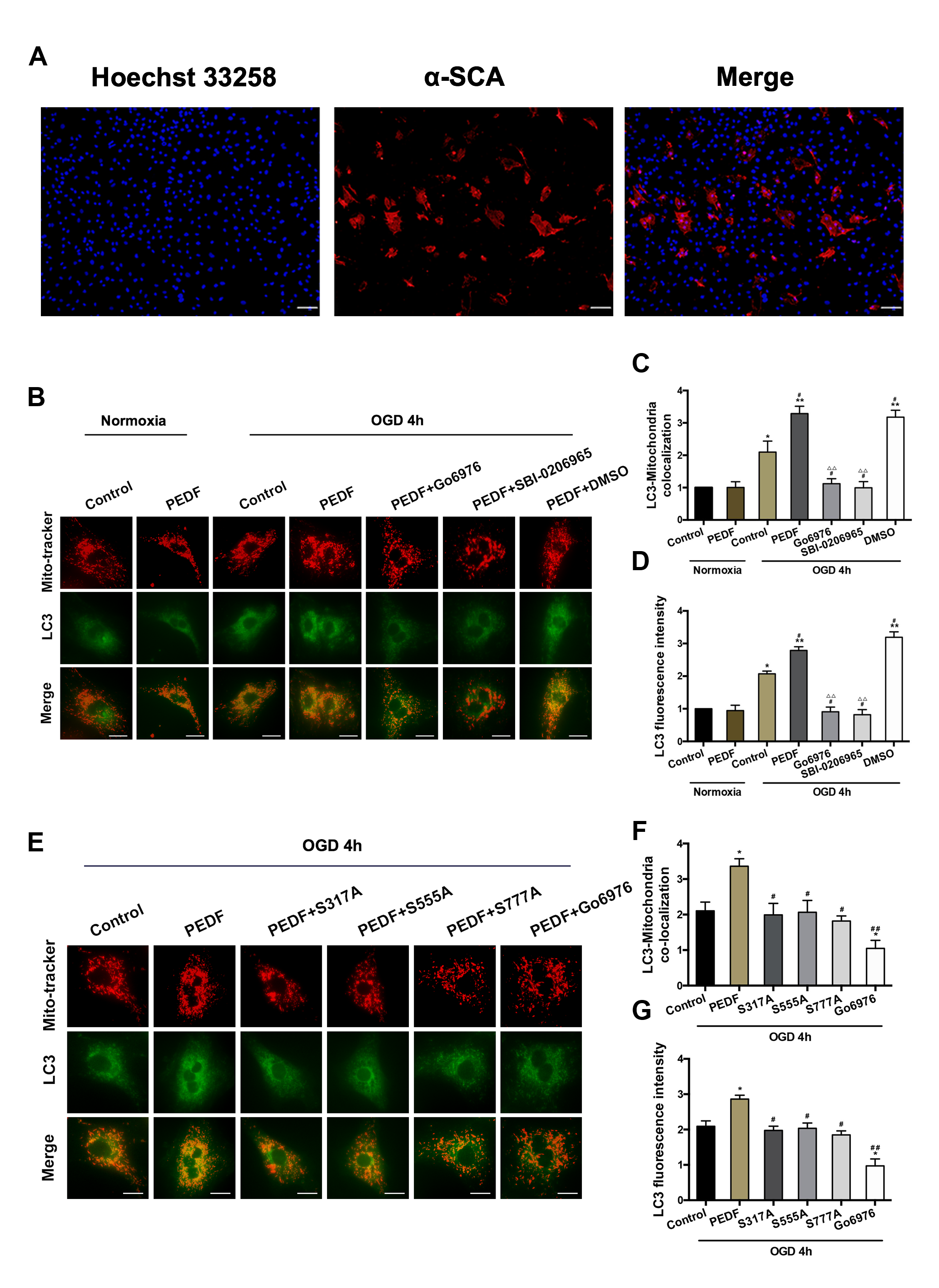


**Figure S1. Confirmation of cardiomyocytes by α-SCA immunofluorescence, and confocal laser scanning microscope of Mito-tracker Red-labeled mitochondria and LC3 staining.**

(A) Immunofluorescence staining of α-sarcomeric actin (α-SCA) to verify cardiomyocytes, bar=100 μm. (B-D) Confocal laser scanning microscope of (B) Mito-tracker Red-labeled mitochondria and LC3 staining and quantification of (C) colocalization rate and (D) fluorescence intensity of LC3/Mito-tracker Red, bar=60 μm. Cardiomyocytes were treated with PEDF, Go6976, SBI-0206965 and DMSO under normal conditions or before OGD for 4 hours, n=30 from 3 independent experiments. (E-G) Confocal laser scanning microscope of (E) Mito-tracker Red-labeled mitochondria and LC3 staining and quantification of (F) colocalization rate and (G) fluorescence intensity of LC3/Mito-tracker Red, bar=60 μm. Cardiomyocytes were treated with PEDF or PEDF+Go6976 before OGD 4 hours, n=30 from 3 independent experiments. *p< 0.05, **p< 0.01 vs. relative normal control, #p< 0.05, ##p<0.01 vs. relative OGD control.


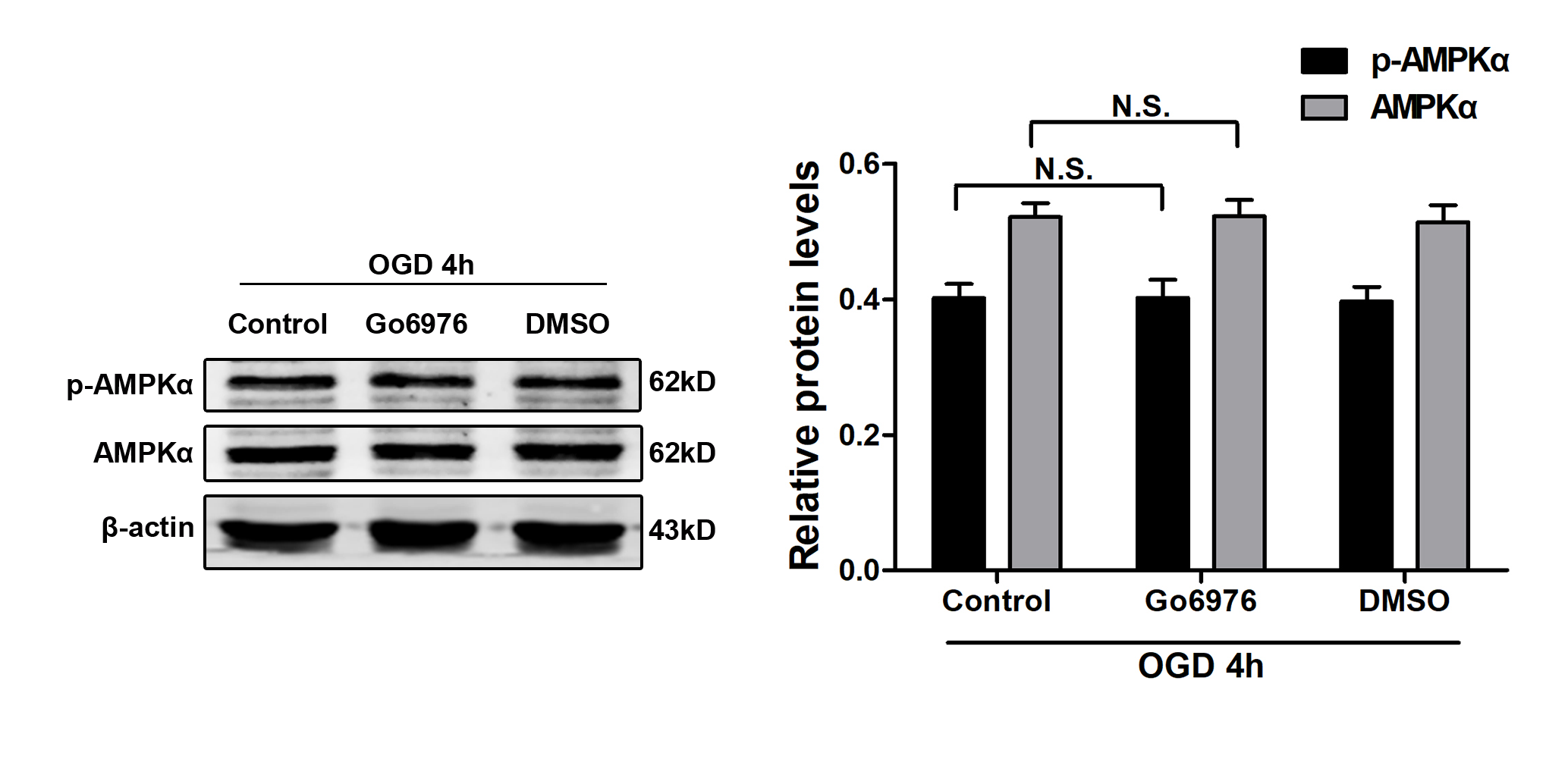


**Figure S2. PKC-inhibitor (Go6976) has no effect on expression of p-AMPK and AMPK in OGD condition.**

Protein levels of p-AMPKα, AMPKα in cardiomyocytes treated with Go6976 or not subjected to OGD for 4 hours were analyzed by western blot, n=4.
